# Inferring a protein interaction map of Mycobacterium tuberculosis based on sequences and interologs

**DOI:** 10.1186/1471-2105-13-S7-S6

**Published:** 2012-05-08

**Authors:** Zhi-Ping Liu, Jiguang Wang, Yu-Qing Qiu, Ross KK Leung, Xiang-Sun Zhang, Stephen KW Tsui, Luonan Chen

**Affiliations:** 1 Key Laboratory of Systems Biology, SIBS-Novo Nordisk Translational Research Centre for PreDiabetes, Shanghai Institutes for Biological Sciences, Chinese Academy of Sciences, Shanghai 200031, China; 2 National Center for Mathematics and Interdisciplinary Sciences, Chinese Academy of Sciences, Beijing 100190, China; 3 Beijing Institute of Genomics, Chinese Academy of Sciences, Beijing 100029, China; 4 Academy of Mathematics and Systems Science, Chinese Academy of Sciences, Beijing 100190, China; 5 Hong Kong Bioinformatics Centre, School of Biomedical Sciences, The Chinese University of Hong Kong, Shatin N. T., Hong Kong, China; 6 Collaborative Research Center for Innovative Mathematical Modelling, Institute of Industrial Science, University of Tokyo, Tokyo 153-8505, Japan

## Abstract

**Background:**

*Mycobacterium tuberculosis *is an infectious bacterium posing serious threats to human health. Due to the difficulty in performing molecular biology experiments to detect protein interactions, reconstruction of a protein interaction map of *M. tuberculosis *by computational methods will provide crucial information to understand the biological processes in the pathogenic microorganism, as well as provide the framework upon which new therapeutic approaches can be developed.

**Results:**

In this paper, we constructed an integrated *M. tuberculosis *protein interaction network by machine learning and ortholog-based methods. Firstly, we built a support vector machine (SVM) method to infer the protein interactions of *M. tuberculosis *H37Rv by gene sequence information. We tested our predictors in *Escherichia coli *and mapped the genetic codon features underlying its protein interactions to *M. tuberculosis*. Moreover, the documented interactions of 14 other species were mapped to the interactome of *M. tuberculosis *by the interolog method. The ensemble protein interactions were validated by various functional relationships, i.e., gene coexpression, evolutionary relationship and functional similarity, extracted from heterogeneous data sources. The accuracy and validation demonstrate the effectiveness and efficiency of our framework.

**Conclusions:**

A protein interaction map of *M. tuberculosis *is inferred from genetic codons and interologs. The prediction accuracy and numerically experimental validation demonstrate the effectiveness and efficiency of our method. Furthermore, our methods can be straightforwardly extended to infer the protein interactions of other bacterial species.

## Background

*M. tuberculosis *which causes tuberculosis affecting lungs and other organs is the second largest cause of death from infectious diseases [1]. An extensive protein-protein interaction (PPI) network of *M. tuberculosis *can lead to more comprehensive screens of cellular operations. In this context, development of approaches to infer its interactome will contribute to identifying infectious mechanisms, detecting important drug target proteins and promoting potential therapy innovations. To date, genome-wide experimental and computational systems for studying PPIs in *M. tuberculosis *are unavailable [2]. It is necessary to develop approaches capable of converting available genomic data into functional information of protein-interaction map for *M. tuberculosis*. *E. coli *is one of the best model systems to study bacterial physiology [3], with relatively well-characterized interactome, genome and transciptome [4]. It is believed that the protein interactions are conserved in different organisms [5]. The interaction features can be learned by machine learning methods, such as support vector machines (SVMs) [6,7], and also it is common to predict protein interactions from the known interactions of other organisms by interolog method [8].

Compared with other methods, sequence-based prediction methods are superior for their simple requirement on the data, which could be implemented when the species have completely sequenced genomes. There were some studies that are based on sequence information have been successfully performed on PPI prediction of some model organisms such as *H. sapiens*, *S. cerevisiae *and *E. coli *[6,9-12]. However, a limitation of these methods is the requirement of large size of training data to meet a satisfactory accuracy criterion. For model organisms, we have a large volume of prior PPIs that can be used as training data, but there are few experimental data of PPI for some dangerous bacteria like *M. tuberculosis*. Thus, a novel integration method is necessary to be developed. In this work, we provided cross-species PPI predictions in *M. tuberculosis *by integrating different types of protein interaction information of other species. Genetic information in the form of codons, i.e. tri-nucleotide sequences, are translated into proteins [13]. It is well known that codon usage is correlated with expression level [9,13]. The codon which carries genetic information specifies the amino acid sequence in the polypeptide during the synthesis of proteins. The genetics of coding sequences is not only the blueprint for translating amino acids, but also the continuous original information for genetic transcription of gene expression. Here, genetic codons will be selected as the sequence features in the learning of interaction patterns. Moreover, the corresponding orthologs of interacting proteins in other organisms will provide more information about the potential interaction mappings by comparative genomics.

In this work, we developed a systematic method combining heterogeneous data sources to infer a comprehensive protein interaction map for *M. tuberculosis*. The codon features of interacting protein pairs are detected and used to train an SVM classifier. Then the interactome of *M. tuberculosis *is predicted by the codon-based method. Moreover, the interactions from 14 other species are mapped to *M. tuberculosis *by the interolog method. The available data from multiple levels including gene coexpression, evolutionary relationship and functional similarity are implemented to assess these predicted interactions by confidence significance. The evidence from various sources validates the effectiveness of our method. The network properties of the constructed protein-interaction map are also identified. The predicted protein interaction network as well as the proposed method provide a framework for the functional specificities study of *M. tuberculosis*.

## Results

### Predictor performance

**Figure 1:**
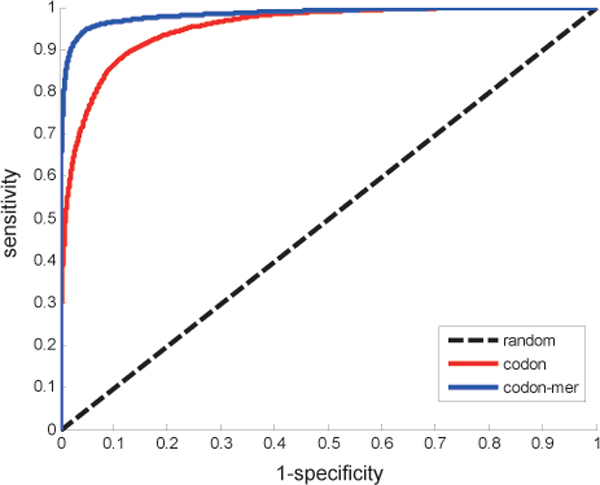
**ROC curves of the five-fold cross validation predictions in E. coli**.

*E. coli *is one of the best characterized organisms [3,4] and we chose it as a model system for building the protein interaction map of *M. tuberculosis*. The positive and negative sets of protein interactions in *E. coli *were designed to test the performance of our codon-based prediction methods. The genome and proteome of *E. coli *were downloaded and prepared for the interacting sets as well as all known opening reading frames (ORFs) [14]. The distance of two ORFs in terms of usage of codon *c *is defined as

$$d_{ij}\left(c\right)=\;|f_{i}\left(c\right)-f_{j}\left(c\right)|,$$

where *f_i_*(*c*) and *f_j_*(*c*) are relative frequencies of codon *c *in ORF *i *and ORF *j*. By codon definition, ∑*_k _f_i_*(*c_k_*) = 1 and ∑*_k _f_j_*(*c_k_*) = 1 for *k *= 1, 2,..., 64 in all codons. There are 14058 pairs of interactions and 27882 pairs of non-interactions in 4227 proteins of *E. coli*. A five-fold cross validation process is implemented in these pairs, i.e., we train the SVM classifier based on the related codons in the 80% interacting pairs forming the training part and test the prediction in the rest part. Figure 1 shows the performance of prediction results of receiver operating characteristic (ROC) curves by the SVM predictor using genetic codon features. As we know, there are several codons corresponding to the same amino acid in genetic code. The prediction performance of merging the frequency of these degenerate codons ('codon-mer') is also shown in Figure 1. The details of prediction precision and accuracy are listed in Table 1. The SVM predictor can achieve the prediction accuracy (ACC) of 0.9003 and the area under curve (AUC) of 0.9507 in the PPI of *E. coli*. These results provide pieces of evidence for the effectiveness and efficiency of predicting protein interactions from the genetic codons by machine learning method.

**Table 1:** Prediction performances of the codon-based SVM predictor in *E. coli*

Feature	ACC	SN	SP	PRE	AUC
Codon	0.9003	0.7576	0.9486	0.8327	0.9507
Codon-mer	0.9595	0.8986	0.9801	0.9386	0.9835

### Protein interactions in M. tuberculosis

**Figure 2:**
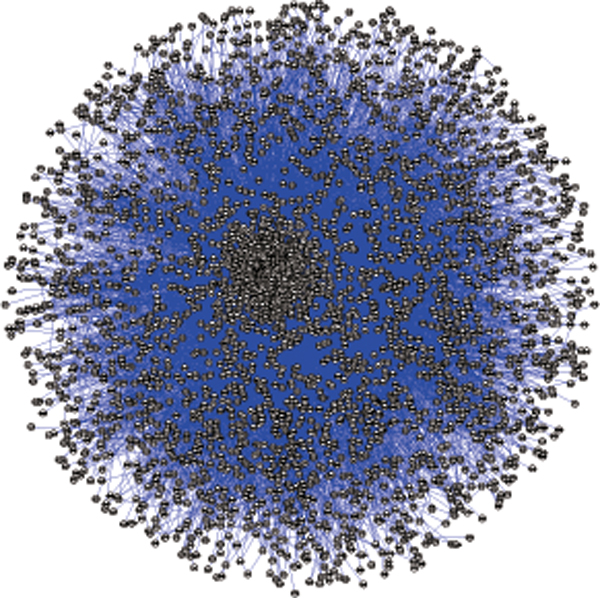
**Inferred protein interaction map in M. tuberculosis**.

To explore protein interactions in *M. tuberculosis*, we used the formerly trained SVM classifier to infer the interactions of *M. tuberculosis *by the codon message of ORFs in gene sequence level. Based on the genetic codons of the laboratory strain H37Rv of *M. tuberculosis*, we predicted 12,899 interactions in 3,266 proteins. Furthermore, the known protein interactions of other species were mapped to the proteome of *M. tuberculosis *by interolog method. We collected the documented interactions of 14 species from PPI databases, IntAct and DIP, and the sequence features of interacting proteins were transferred into the *M. tuberculosis *proteome by ortholog detection. Table 2 lists the detailed prediction results by interolog method. So far, we also found 530 pairs of protein interactions of *M. tuberculosis *from various databases, such as BIND [15] and Reactome [16]. Combining with these known interactions, we built a comprehensive protein interaction map totally with 46,119 interactions of 3,465 proteins in *M. tuberculosis*. The inferred protein interaction map of *M. tuberculosis *is shown in Figure 2.

**Table 2:** Details of predicted protein interactions in *M. tuberculosis*

Species	Database	Original PPI	Predicted PPI	Percentage (%)
By machine learning				

E. coli	ECID	14,058(positive)+ 27,882(negative)	12,899	27.97

By interolog				

Escherichia coli	IntAct	14,158	16,468	35.71
Campylobacter jejuni	IntAct	11,870	7,674	16.64
Treponema pallidum	IntAct	3,744	324	0.70
Synechocystis	IntAct	2,625	2,481	5.38
Myxococcus xanthus	IntAct	384	253	0.55
Synechocystis sp.	IntAct	219	220	0.48
Rickettsia sibirica	IntAct	282	24	0.05
Streptococcus pneumoniae	IntAct	193	47	0.10
Drosophila melanogaster	DIP	22,650	1,558	3.38
Saccharomyces cerevisiae	DIP	21,769	2,701	5.86
Caenorhabditis elegans	DIP	3,979	229	0.50
Homo sapiens	DIP	1,485	84	0.18
Mus musculus	DIP	287	36	0.06
Rattus norvegicus	DIP	69	2	0.15

Total: 46,119 interactions in 3,465 proteins (with 530 known PPIs)				

### Validation results

Interacting protein pairs have been identified with close relationship of gene coexpression [17], coevolution [18], similar GO annotations [19], phenotype association and similar physicochemical elements [20]. For *M. tuberculosis *species, we got these available heterogeneous data sources to annotate every predicted interacting pairs.

**Figure 3:**
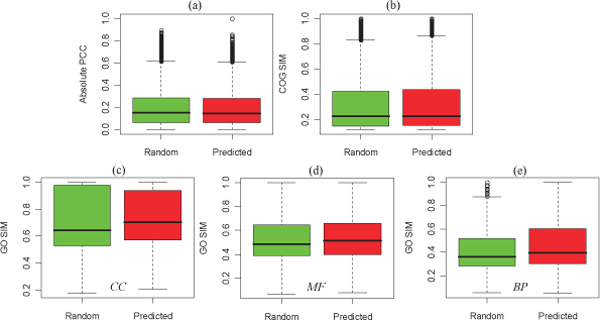
**Boxplot of coexpression (a), coevoluation (b) and cofunction values (c)-(e) of the predicted interactions and that of the same-size random selected protein pairs**.

Firstly, we annotated the predicted interacting protein pairs by their corresponding Pearson's correlation coefficient (PCC) of gene coexpression. For comparison, we calculated the corresponding correlation values of these same-size random selected protein pairs. Every prediction was then annotated by a coexpression value in gene expression profiling. Figure 3(a) shows the boxplot of coexpression values in the predictions. From Figure 3(a), we identified that the coexpression values in the predicted interacting pairs tend to be more correlated when compared to the same-size randomly selected pairs (*P-value *= 4.69 × 10^-3^, Mann-Whitney U test). Secondly, we identified the evolutionary relationship of the interacting proteins by the clusters of orthologous group (COG) information. The interacting proteins were detected in their own COG individually. Figure 3(b) shows the boxplot of evolutionary relationship values in the predicted interacting pairs and that of the same-size randomly selected protein pairs. Their difference measured by the Mann-Whitney U test (*P-value *= 0.53) is not significant, while every predicted interaction gets a confidence value of evolutionary relationship. Thirdly, we calculated the functional similarities underlying these predicted interactions. We detected the semantic similarity between the gene ontology (GO) term pairs of interacting proteins. We have considered the hierarchical structure of GO directed acyclic graph and the specificity of GO terms in the identification. We identified the functional relationships between predicted interacting proteins by three ontology categories, i.e. cellular component ('CC'), molecular function ('MF') and biological process ('BP'), respectively. The boxplots of the three values of GO similarities in the random pairwise proteins and that in predicted pairs are shown in Figure 3(c), (d) and 3(e), respectively. The predicted interactions have higher values of functional similarity than random ones (*P-value*s are 6.63 ×10^-4^, < 2.2 ×10^-16 ^and < 2.2 ×10^-16^, respectively), which further provides evidence for the effectiveness of our methods. After annotating from various information, we provide the evaluation confidence to every predicted interactions.

### Network analysis

For global views of the protein-interaction map, we identified the topological features of the integrated protein interaction map and the features of particular interactions. Firstly, we detected the original features in the primary constructed network by machine learning and interologs combined with the known interacting protein pairs. The measures of degree distributions, clustering coefficients, characteristic shortest path and network diameter are identified individually. Table 3 lists some network properties. Network diameter is the longest path between any two proteins. The characteristic path length is calculated by averaging minimum distance between protein pairs. Clustering coefficient is a measure of degree to which nodes in a network tend to cluster together [21]. A network whose degree distribution follows a power law is often called a scale-free network [22]. These measures refer to the details of the properties of the inferred protein-interaction map of *M. tuberculosis*. The hub proteins as well as interested proteins can be selected to analyze for particular dysfunctions of *M. tuberculosis*. From the validations of gene coexpression, evolutionary relationship in COGs and functional similarity, we can check and filter out those pairs consistently included in various level information by evaluating the reliability of interactions. We then calculated the features in the filtered network by omitting the pairs with lower confidence values, while we kept the predictions when there are no available evaluations for them. We also identified the distribution of node degree and found that the constructed protein interaction network satisfied the topology features of complex networks [22]. The processes are based on the network analysis of fitting the distribution of a scale-free network, and the parameter *γ *value is asymptotically in the range 1 <*γ *< 2 in the power-law distribution fitting. There are 477 hub proteins in the protein interaction map when the degree threshold is 50. The hub proteins from different thresholds can be found in Additional file 1.

**Table 3:** Topological parameters of protein-interaction map in *M. tuberculosis*

Threshold	Node	Edge	Diameter	Characteristic path length	Clustering coefficient	Power law fitting
None	3465	46119	8	3.341	0.093	1.237
0.5 (PCC)	3409	35582	8	3.419	0.074	1.310
0.5 (COG)	3397	43425	8	3.445	0.099	1.220
0.5 (GOCC)	3461	45193	8	3.355	0.091	1.245
0.5 (GOMF)	3400	31045	8	3.632	0.067	1.431
0.5 (GOBP)	3373	26200	9	3.713	0.076	1.440
0.5 + 0.5 + 0.3	3383	26827	9	3.730	0.062	1.522
(GOCC + GOMF + GOBP)						
0.5 + 0.7	3324	33134	9	3.616	0.080	1.289
(PCC + COG)						
0.5 + 0.9 + 0.8	3304	29891	9	3.691	0.072	1.336
(PCC + COG + GOCC)						
0.5 + 0.7 + 0.4	3212	13244	9	4.388	0.044	1.803
(PCC + GOMF + GOBP)						
0.5 + 0.7	3281	21240	11	3.920	0.081	1.414
(COG + GOBP)						

## Discussions

In this work, we proposed a method to build the protein-interaction map in *M. tuberculosis *by machine learning and interologs. We obtained the interaction features of genetic codon underlying interacting proteins in relatively well-established interactome of *E. coli*. The features of genetic codons of interacting proteins of *E. coli *were mapped to the proteome of *M. tuberculosis *by training an SVM classifier. The cross validation showed the effectiveness and efficiency of our predictor. We also implemented the interolog method to map the documented protein interactions of other organisms into *M. tuberculosis*. Moreover, the available functional genomic information about *M. tuberculosis *has been used to evaluate the predicted interactions. These heterogeneous data were combined in a novel framework to infer the interactions in *M. tuberculosis*. The predicted pairs were checked and can be filtered with these information for potential applications. The constructed protein interaction network of *M. tuberculosis *provides more information for the infectious bacterium threatening human health.

We used multiple sources of available functional genomic data to provide evaluation of these predicted interactions. Gene coexpression, evolutionary relationship and functional similarity are implemented to check the reliability in the targeted pairs. The information could be directly used to build the functional relationship of protein pairs [23-25]. Due to the limited knowledge in *M. tuberculosis*, we integrated the heterogeneous information in an alternative framework for assessing the predictions rather than predicting the interactions. Filtering interactions by different confidence values result in different networks of different size and reliability. This will provide valuable resources for biological information in tuberculosis research, which implies the promising applications based on our constructed protein interaction map, which are our future research topics.

**Figure 4:**
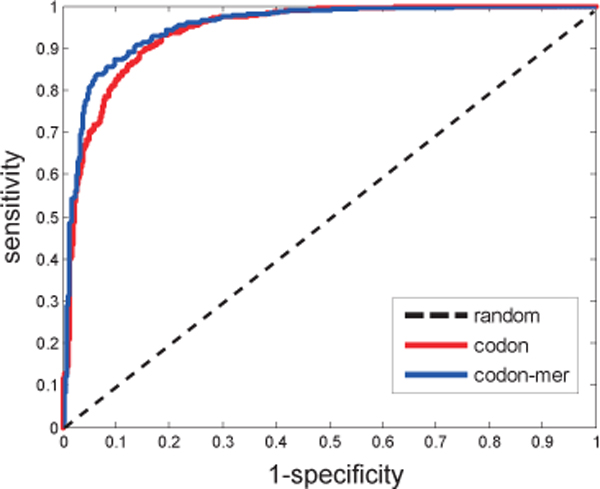
**ROC curves of predicting performance in the known interactions in M. tuberculosis**.

In our framework, we proposed a cross-species prediction by mapping the documented interactions of other species into *M. tuberculosis*. For completeness, we collected some known curated interactions. We also tested the predictions in these known interactions in *M. tuberculosis *by training the codon features to check our predictions. Figure 4 shows the ROC curves of prediction performance. Our method can achieve high AUC of 0.945 by the codon-based method and of 0.951 by merging the frequency of these degenerate codons in these known protein interactions. Table 4 shows the results of prediction performances. We achieved similar accuracy by merging the codons as that by the codon-based method. In our previous work, we concluded that there is subtle difference between the two encoding schemes for predicting protein interactions [7]. Both methods are rational and their differences are underlying the data sets. The results provide more evidence for the effectiveness and efficiency of our proposed methods.

**Table 4:** Prediction performances of the SVM predictor in these known protein interactions of *M. tuberculosis*

Feature	ACC	SN	SP	PRE	AUC
Codon	0.8728	0.8932	0.8524	0.8582	0.9454
Codon-mer	0.8738	0.8971	0.8505	0.8571	0.9507

Basically, we implemented two pipelines of building the protein-interaction map of *M. tuberculosis*, i.e., the SVM-based machine learning method and the interolog mapping method. The two methods are essentially close-related. The gene sequence information of interacting pair of proteins has been learned by the predictor and that of these known interactions is mapped to the protein pairs of *M. tuberculosis*. In the same manner, the interolog method identifies the interaction between a pair of proteins which have interacting homologs in another organism. The protein sequence information of known interaction is mapped by the cross-species sequence similarity detection. It is an interesting research topic to identify the quantitative relationship between the prediction results of the two methods. The various mapping schemes of the sequence information have been integrated in our predictions. The gene sequence information as well as the protein sequence information is exploited to infer the protein-interaction map of *M. tuberculosis*. The other research direction is to implement other schemes to encode the sequence information in the machine learning method, such as the autocorrelation encoding scheme [26] and triplet residues method [6]. We combined the gene sequence information and the protein sequence information into an integrated framework. It is also an interesting topic to investigate the prediction difference of the two-level sequence information.

## Conclusion

In conclusion, we provided a novel framework to integrate genomic data to infer a protein interaction map of *M. tuberculosis*. We predicted the protein interactions in *M. tuberculosis *by an SVM based classifier by genetic codons. And the documented protein interactions from various species were also mapped to the proteome of *M. tuberculosis *by interolog method. The information from gene expression, evolutionary and functional relationship provided reliability measures of evaluating our predictions. The validations provided clear evidence for the effectiveness of our method. Our framework can easily be extended to infer the large-scale protein interaction map in other species. These predicted interactions provide a valuable reference of interactome for *M. tuberculosis *research. The PPIs build a frame to further study the functional implications underlying the interactome of *M. tuberculosis*. They are listed in Additional file 2. The details are available at: http://www.aporc.org/doc/wiki/MTBPPI.

## Methods

### Framework of prediction

**Figure 5:**
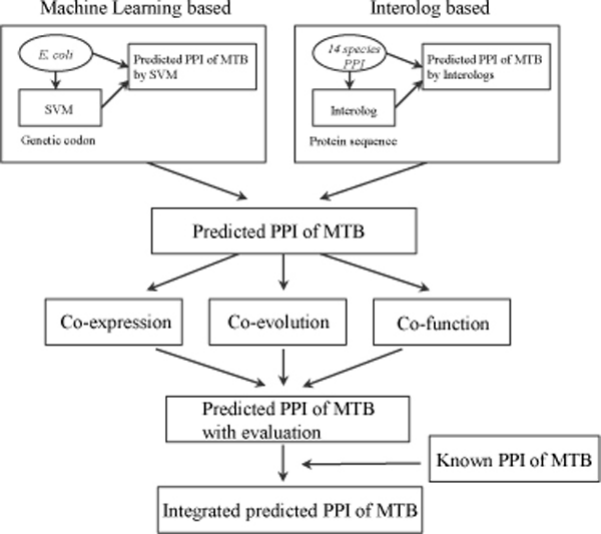
**Framework of inferring protein interaction map in M. tuberculosis**.

Figure 5 shows our framework to infer the protein interaction map of *M. tuberculosis*. The protein interactions were predicted by two main pipelines. Firstly, we built the protein interaction network of *M. tuberculosis *from codon features of interacting proteins in *E. coli *by a machine learning approach. The integrated interaction map and gene sequences of *E. coli *were downloaded from EcID, which collects comprehensive PPIs in *E. coli *by combining various knowledge [4]. We used the information of protein interactions of *E. coli *to train an SVM classifier to get the genetic codon features underlying these interacting pairs. The interactions in *M. tuberculosis *were then predicted by the trained SVM predictor with the genetic codons of ORFs in gene sequences of *M. tuberculosis*. We chose the laboratory strain of H37Rv as our model organism [14]. The processes are shown in the upper-left square frame of Figure 5. Secondly, we inferred the protein interactions of *M. tuberculosis *by interolog method from the documented protein interactions in 14 other species. We collected these interactions from IntAct [27] and DIP [28]. The interacting proteins of each species were detected their homologous proteins of *M. tuberculosis *by BLAST [29] individually. The homologs of two interacting proteins will be identified as the predicted interactors. The pipeline is shown in the upper-right square framework of Figure 5. As for the validation of predicted results, we tested our method in *E. coli *and in the known interactions of *M. tuberculosis*. Three pieces of available information of *M. tuberculosis*, i.e., gene expression profiling, evolutionary relationship from ortholog database and functional similarity, were used to evaluate the confidence of prediction results. The known protein interactions were of course included in our constructed interactome of *M. tuberculosis*. Finally, we inferred an integrated protein interaction map of *M. tuberculosis*.

### SVM-based predictor

We used the SVM method [30] as the classifier. The software libsvm 2.84 [31] was employed and a radial basis function was chosen as the kernel function in our implementation. The positive pairs of training are those known interactions which are experimentally validated in EcID. There were 14,058 pairs of positive interactions. We selected the negative set by choosing the pairs when the length of shortest path between the two terminals in EcID network is larger than a given cutoff of 6 for the small-world property of a complex network [21]. There is few possibility for two proteins to interact with each other when the distance is bigger than the threshold. There were 27,882 pairs of proteins which are included in the negative set. A five-fold cross validation process was implemented to test the accuracy of our SVM-based classifier. We applied the trained predictor to infer the protein interactions in *M. tuberculosis*.

The prediction performance was evaluated by various parameters, such as sensitivity (SN), specificity (SP), accuracy (ACC) and precision (PRE). The evaluation is usually displayed in a ROC graph with measure of area under curve (AUC). Mathematically, these measures are defined as

$$\begin{array}{c} SN=\frac{TP}{TP+FN},\\ SP=\frac{TN}{TN+FP},\\ ACC=\frac{TP+TN}{TP+TN+FP+FN},\\ PRE=\frac{TP}{TP+FP}, \end{array}$$

where TP, TN, FP and FN refer to number of true positive, number of true negative, number of false positive, and number of false negative predictions, respectively.

### Validation from multiple resources

We constructed the protein interaction map of *M. tuberculosis *by genetic codons and ortholog mapping. We also deposited known interactions in databases from experimental results about *M. tuberculosis *in literatures. Integrated with these known protein interactions, we built a comprehensive PPI map of *M. tuberculosis*. We collected multiple available resources to access the constructed protein interaction map in *M. tuberculosis*. The confidence of interactions was evaluated by three extra data sources, namely, gene expression, evolutionary relationship and functional similarity.

**Figure 6:**
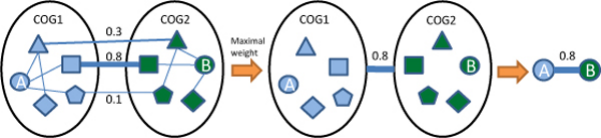
**Identification of evolutionary relationship between two interacting proteins**. The maximum of COG value between two groups in which the interacting proteins are located are used as the value of their evolutionary relationship.

Firstly, we identified the PCCs of gene coexpression of pairwise proteins in the predicted network. We downloaded the gene expression profiling data of *M. tuberculosis *H37Rv from NCBI GEO (ID: GSE9776) [32]. Correlation between genes is calculated by

$$cor\left(x_{i},x_{j}\right)=\frac{E\left(x_{i}-\mu_{x_{i}}\right)\left(x_{j}-\mu_{x_{j}}\right)}{\sigma_{i}\sigma_{j}},$$

where $\mu_{x_{i}}$ and $\mu_{x_{j}}$ are the means of gene expression profile *x_i _*and *x_j_*, *σ_i _*and *σ_j _*are the standard deviations of them. Secondly, we presented the evaluation of evolutionary relationship between the predicted interacting proteins. COGs were delineated by comparing protein sequences encoded in complete genomes, representing major phylogenetic lineages [33]. Figure 6 presents the method to identify the evolutionary information between the predicted interacting proteins. Each COG consists of individual proteins or groups paralogs from at least 3 lineages and thus corresponds to an ancient conserved domain [33]. The maximum of COG value between two groups in which the interacting proteins are located were regarded as the value representing their evolutionary relationship. Thirdly, GO [34] similarity between the predicted pairs was identified to evaluate their functional relationship. We downloaded the annotations for *M. tuberculosis *H37Rv from GOA [35]. In GO hierarchical acyclic graph, the terms far from the root would be more informative than those close to the root. We calculated the GO probability for specific GO terms [36]. The frequency of a GO term in a database is defined as

$$freq\left(c\right)=anno\left(c\right)+\underset{h\in children\left(c\right)}{\sum }freq\left(h\right),$$

where *anno*(*c*) is the number of proteins annotated with this terms in our database. The set of child nodes of term *c *is the *children*(*c*). The probability of a term *t *is then defined as *p*(*c*) = *freq*(*c*)/*freq*(*root*), where *freq*(*root*) is the frequency of the root term [37,38]. We used semantic similarity measures [36-38] to evaluate the similarity of GO term lists corresponding to the interacting proteins. Based on these validations, we can check those interactions consistently validated in various information and detect an ensemble protein network by omitting low reliability pairs.

## Abbreviations

PPI: protein-protein interaction; SVM: support vector machine; ROC: receiver operating characteristic; AUC: area under curve; ACC: accuracy; SP: specificity; SN: sensitivity; PRE: precision; TP: true positive; TN: true negative; FP: false positive; FN: false negative; BIND: biomolecular interaction network database; BioGrid: biological general repository for interaction datasets; IntAct: molecular interaction database; ORF: opening reading frame; COG: cluster of orthologous group; GO: gene ontology; CC: cellular component; MF: molecular function; BP: biological process; NCBI: national center for biotechnology information; GEO: gene expression omnibus.

## Competing interests

The authors declare that they have no competing interests.

## Authors' contributions

ZPL, JW and LC conceived the research. ZPL and JW designed and performed the study. YQQ, RKKL, XSZ and SKWT gave valuable suggestions and improvements. LC supervised the project. ZPL wrote the paper with contributions from others. All authors read and approved the manuscript.

## Supplementary Material

Additional file 1 **Hub proteins in the protein interaction map of M. tuberculosis**. Click here for file

Additional file 2 **The protein interaction map of M. tuberculosis**. Click here for file

## References

[B1] Reddy T Riley R Wymore F Montgomery P DeCaprio D Engels R Gellesch M Hubble J Jen D Jin H TB database: an integrated platform for tuberculosis research Nucleic Acids Res 2009 37 D499 D508 10.1093/nar/gkn652 18835847 PMC2686437

[B2] Singh A Mai D Kumar A Steyn A Dissecting virulence pathways of Mycobacterium tuberculosis through protein-protein association Proc Natl Acad Sci USA 2006 103 11346 11351 10.1073/pnas.0602817103 16844784 PMC1544089

[B3] Arifuzzaman M Maeda M Itoh A Nishikata K Takita C Saito R Ara T Nakahigashi K Huang H Hirai A Large-scale identification of protein-protein interaction of Escherichia coli K-12 Genome Res 2006 16 686 691 10.1101/gr.4527806 16606699 PMC1457052

[B4] Andres Leon E Ezkurdia I García B Valencia A Juan D EcID. A database for the inference of functional interactions in E. coli Nucleic Acids Res 2009 37 D629 D635 10.1093/nar/gkn853 19004873 PMC2686479

[B5] Hirsh E Sharan R Identification of conserved protein complexes based on a model of protein network evolution Bioinformatics 2007 23 e170 e176 10.1093/bioinformatics/btl295 17237088

[B6] Shen J Zhang J Luo X Zhu W Yu K Chen K Li Y Jiang H Predicting protein-protein interactions based only on sequences information Proc Natl Acad Sci USA 2007 104 4337 4341 10.1073/pnas.0607879104 17360525 PMC1838603

[B7] Wang Y Wang J Yang Z Deng N Sequence-based protein-protein interaction prediction via support vector machine J Syst Sci & Complexity 2010 23 1012 1023

[B8] Yu H Luscombe N Lu H Zhu X Xia Y Han J Bertin N Chung S Vidal M Gerstein M Annotation transfer between genomes: protein-protein interologs and protein-DNA regulogs Genome Res 2004 14 1107 1118 10.1101/gr.1774904 15173116 PMC419789

[B9] Najafabadi H Salavati R Sequence-based prediction of protein-protein interactions by means of codon usage Genome Biol 2008 9 R87 10.1186/gb-2008-9-5-r87 18501006 PMC2441473

[B10] Xia J Zhao X Huang D Predicting protein-protein interactions from protein sequences using meta predictor Amino Acids 2010 39 1595 1599 10.1007/s00726-010-0588-1 20386937

[B11] You Z Lei Y Huang D Zhou X Using manifold embedding for assessing and predicting protein interactions from high-throughput experimental data Bioinformatics 2010 26 2744 2751 10.1093/bioinformatics/btq510 20817744 PMC3025743

[B12] Shi M Xia J Li X Huang D Predicting protein-protein interactions from sequence using correlation coefficient and high-quality interaction dataset Amino Acids 2010 38 891 899 10.1007/s00726-009-0295-y 19387790

[B13] Jansen R Bussemaker H Gerstein M Revisiting the codon adaptation index from a whole-genome perspective: analyzing the relationship between gene expression and codon occurrence in yeast using a variety of models Nucleic Acids Res 2003 31 2242 2251 10.1093/nar/gkg306 12682375 PMC153734

[B14] Cole S Brosch R Parkhill J Garnier T Churcher C Harris D Gordon S Eiglmeier K Gas S Barry C Deciphering the biology of Mycobacterium tuberculosis from the complete genome sequence Nature 1998 393 537 544 10.1038/31159 9634230

[B15] Alfarano C Andrade C Anthony K Bahroos N Bajec M Bantoft K Betel D Bobechko B Boutilier K Burgess E The Biomolecular Interaction Network Database and related tools - 2005 update Nucleic Acids Res 2005 33 D418 D424 15608229 10.1093/nar/gki051 PMC540005

[B16] Vastrik I D'Eustachio P Schmidt E Joshi-Tope G Gopinath G Croft D de Bono B Gillespie M Jassal B Lewis S Matthews L Wu G Birney E Stein L Reactome: a knowledge base of biologic pathways and processes Genome Biol 2007 8 R39 10.1186/gb-2007-8-3-r39 17367534 PMC1868929

[B17] Jansen R Greenbaum D Gerstein M Relating whole-genome expression data with protein-protein interactions Genome Res 2002 12 37 46 10.1101/gr.205602 11779829 PMC155252

[B18] Jothi R Kann M Przytycka T Predicting protein-protein interaction by searching evolutionary tree automorphism space Bioinformatics 2005 21 i241 i250 10.1093/bioinformatics/bti1009 15961463 PMC1618802

[B19] Mahdavi M Lin Y False positive reduction in protein-protein interaction predictions using gene ontology annotations BMC Bioinformatics 2007 8 262 10.1186/1471-2105-8-262 17645798 PMC1941744

[B20] Chen L Wu L Wang Y Zhang X Inferring protein interactions from experimental data by association probabilistic method Proteins 2006 62 833 837 10.1002/prot.20783 16395667

[B21] Albert R Barabasi A Statistical mechanics of complex networks Reviews of Modern Physics 2002 74 47 10.1103/RevModPhys.74.47

[B22] Barabasi A Oltvai Z Network biology: understanding the cell's functional organization Nat Rev Genet 2004 5 101 113 10.1038/nrg1272 14735121

[B23] Eisenberg D Marcotte E Xenarios I Yeates T Protein function in the post-genomic era Nature 2000 405 823 826 10.1038/35015694 10866208

[B24] Jansen R Yu H Greenbaum D Kluger Y Krogan N Chung S Emili A Snyder M Greenblatt J Gerstein M A Bayesian networks approach for predicting protein-protein interactions from genomic data Science 2003 302 449 453 10.1126/science.1087361 14564010

[B25] Lee I Date S Adai A Marcotte E A probabilistic functional network of yeast genes Science 2004 306 1555 1558 10.1126/science.1099511 15567862

[B26] Guo Y Yu L Wen Z Li M Using support vector machine combined with auto covariance to predict protein-protein interactions from protein sequences Nucleic Acids Res 2008 36 3025 3030 10.1093/nar/gkn159 18390576 PMC2396404

[B27] Kerrien S Alam-Faruque Y Aranda B IntAct-open source resource for molecular interaction data Nucleic Acids Res 2007 35 D561 D565 10.1093/nar/gkl958 17145710 PMC1751531

[B28] Xenarios I Salwinski L Duan X Higney P Kim S Eisenberg D DIP, the Database of Interacting Proteins: a research tool for studying cellular networks of protein interactions Nucleic Acids Res 2002 30 303 305 10.1093/nar/30.1.303 11752321 PMC99070

[B29] Altschul S Madden T Schaffer A Zhang J Zhang Z Miller W Lipman D Gapped BLAST and PSI-BLAST: a new generation of protein database search programs Nucleic Acids Res 1997 25 3389 3402 10.1093/nar/25.17.3389 9254694 PMC146917

[B30] Vapnik V The Nature of Statistical Learning Theory 1995 New York: Springer-Verlag

[B31] Chang C Lin C LIBSVM: a library for support vector machines ACM Transactions on Intelligent Systems and Technology 2011 2 1 27

[B32] Barrett T Troup D Wilhite S Ledoux P Rudnev D Evangelista C Kim I Soboleva A Tomashevsky M Edgar R NCBI GEO: mining tens of millions of expression profiles-database and tools update Nucleic Acids Res 2007 35 D760 D765 10.1093/nar/gkl887 17099226 PMC1669752

[B33] Tatusov R Galperin M Natale D Koonin E The COG database: a tool for genome-scale analysis of protein functions and evolution Nucleic Acids Res 2000 28 33 36 10.1093/nar/28.1.33 10592175 PMC102395

[B34] Gene Ontology Consortium Gene ontology: tool for the unification of biology Nat Genet 2000 25 25 29 10.1038/75556 10802651 PMC3037419

[B35] Camon E Magrane M Barrell D Lee V Dimmer E Maslen J Binns D Harte N Lopez R Apweiler R The Gene Ontology Annotation (GOA) Database: sharing knowledge in Uniprot with Gene Ontology Nucleic Acids Res 2004 32 D262 D266 10.1093/nar/gkh021 14681408 PMC308756

[B36] Lord P Stevens R Brass A Goble C Investigating semantic similarity measures across the Gene Ontology: the relationship between sequence and annotation Bioinformatics 2003 19 1275 1283 10.1093/bioinformatics/btg153 12835272

[B37] Schlicker A Domingues F Rahnenfuhrer J Lengauer T A new measure for functional similarity of gene products based on Gene Ontology BMC Bioinformatics 2006 7 302 10.1186/1471-2105-7-302 16776819 PMC1559652

[B38] Liu ZP Wu LY Wang Y Chen L Zhang XS Predicting gene ontology functions from protein's regional surface structures BMC Bioinformatics 2007 8 475 10.1186/1471-2105-8-475 18070366 PMC2233648

